# Lateral flow assay (LFA) in the diagnosis of COVID-19-associated pulmonary aspergillosis (CAPA): a single-center experience

**DOI:** 10.1186/s12879-022-07828-y

**Published:** 2022-11-08

**Authors:** Istemi Serin, Sevim Baltali, Tahir Alper Cinli, Hasan Goze, Burçak Demir, Osman Yokus

**Affiliations:** 1 Department of Hematology, Istanbul Training and Research Hospital, University of Health Sciences, Org. Nafiz GURMAN Cad. 34098, Fatih, Istanbul, Turkey; 2 Department of Anesthesiology and Reanimation, Istanbul Training and Research Hospital, University of Health Sciences, Istanbul, Turkey

**Keywords:** COVID-19-associated pulmonary aspergillosis (CAPA), Lateral flow assay (LFA), Sensitivity, Specificity, Prognosis

## Abstract

**Background:**

Invasive pulmonary aspergillosis (IPA) is seen during coronavirus-2019 (COVID-19), has been reported in different incidences, and is defined as COVID-19-associated pulmonary aspergillosis (CAPA). Detection of galactomannan antigen is an important diagnostic step in diagnosing IPA. Enzyme-linked immunoassay (ELISA) is the most frequently used method, and lateral flow assay (LFA) is increasingly used with high sensitivity and specificity for rapid diagnosis. The present study aimed to compare the sensitivity of LFA and ELISA in the diagnosis of CAPA in COVID-19 patients followed in our hospital's ICU for pandemic (ICU-P).

**Methods:**

This study included patients with a diagnosis of COVID-19 cases confirmed by polymerase chain reaction and were followed up in ICU-P between August 2021 and February 2022 with acute respiratory failure. The diagnosis of CAPA was based on the European Confederation of Medical Mycology (ECMM) and the International Society for Human and Animal Mycology 2020 (ECMM/ ISHAM) guideline. Galactomannan levels were determined using LFA and ELISA in serum samples taken simultaneously from the patients.

**Results:**

Out of the 174 patients followed in the ICU-P, 56 did not meet any criteria for CAPA and were excluded from the analysis. The rate of patients diagnosed with proven CAPA was 5.7% (10 patients). A statistically significant result was obtained with LFA for the cut-off value of 0.5 ODI in the diagnosis of CAPA (p < 0.001). The same significant statistical relationship was found for the cut-off value of 1.0 ODI for the ELISA (p < 0.01). The sensitivity of LFA was 80% (95% CI: 0.55–1.05, p < 0.05), specificity 94% (95% CI: 0.89–0.98, p < 0.05); PPV 53% (95% CI: 0.28–0.79, p > 0.05) and NPV was 98% (95% CI: 0.95–1.01, p < 0.05). The risk of death was 1.66 (HR: 1.66, 95% CI: 1.02–2.86, p < 0.05) times higher in patients with an LFA result of ≥ 0.5 ODI than those with < 0.5 (p < 0.05).

**Conclusions:**

It is reckoned that LFA can be used in future clinical practice, particularly given its effectiveness in patients with hematological malignancies and accuracy in diagnosing CAPA.

## Background

Coronavirus-2019 (COVID-19) continues to maintain its agenda as a serious pandemic factor. Different clinical manifestations associated with COVID-19 challenge clinicians, and secondary infections are an important cause of morbidity. Secondary bacterial infections have increased the need for mechanical ventilation and mortality [1, 2]. Invasive fungal infections occur as another cause of morbidity in the course of COVID-19 [3–6].

Invasive pulmonary aspergillosis (IPA), defined as COVID-19-associated pulmonary aspergillosis (CAPA), has been reported in different incidences [7–16]. IPA is one of the viral infections increasing susceptibility to bacterial and fungal infections [5, 6]. Respiratory viruses can directly damage the airway and predispose to fungal infections [5, 6]. Lympopenia, especially seen in the course of viral infections, is an important risk factor [5, 6]. In addition, steroid use has been identified as an important risk factor for both IPA susceptibility and mortality [4, 7]. Conditions that cause profound immunosuppression such as hematopoietic stem cell transplantation or acute leukemias or conditions associated with respiratory epithelial damage such as chronic obstructive pulmonary disease or asthma are the main predisposing diseases [5–8]. Broad-spectrum antibiotic therapy agents used in intensive care units (ICUs) also disrupt the natural microbial barriers and increase susceptibility to fungal infections [4, 7]. Hypertension, coronary heart diseases, and diabetes are the other defined predisposing diseases for CAPA [4]. *Aspergillus fumigatus* was found to be the most prevalent *Aspergillus* spp. isolated among respiratory samples with positive cultures followed by *Aspergillus flavus* [17].

Detection of galactomannan antigen is an important diagnostic step in diagnosing IPA. Galactomannan is a polysaccharide antigen located in the wall structure of *Aspergillus* species [18]. Enzyme-linked immunoassay (ELISA) is the most frequently used method, and lateral flow assay (LFA) is increasingly used with high sensitivity and specificity for rapid diagnosis. LFA is a self-contained immunochromatographic test for the qualitative and quantitative detection of galactomannan antigen from different samples. The principle of LFA use is based on the lateral flow, and it is based on the formation of complexes of galactomannan-specific antibodies in samples with antigens. The resulting complex is detected with a visible line, and quantitative results are obtained utilizing different specific devices [19]. In a study from our clinic, the diagnostic efficiency of LFA and ELISA were evaluated simultaneously for 87 patients diagnosed with hematological malignancy. LFA was more specific than ELISA in terms of the cut-off value of 0.5 optical density index (ODI) [20].

The present study aimed to compare the sensitivity of LFA and ELISA in the diagnosis of CAPA in COVID-19 patients followed in our hospital's ICU for pandemic (ICU-P).

## Materials and methods

**Fig. 1:**
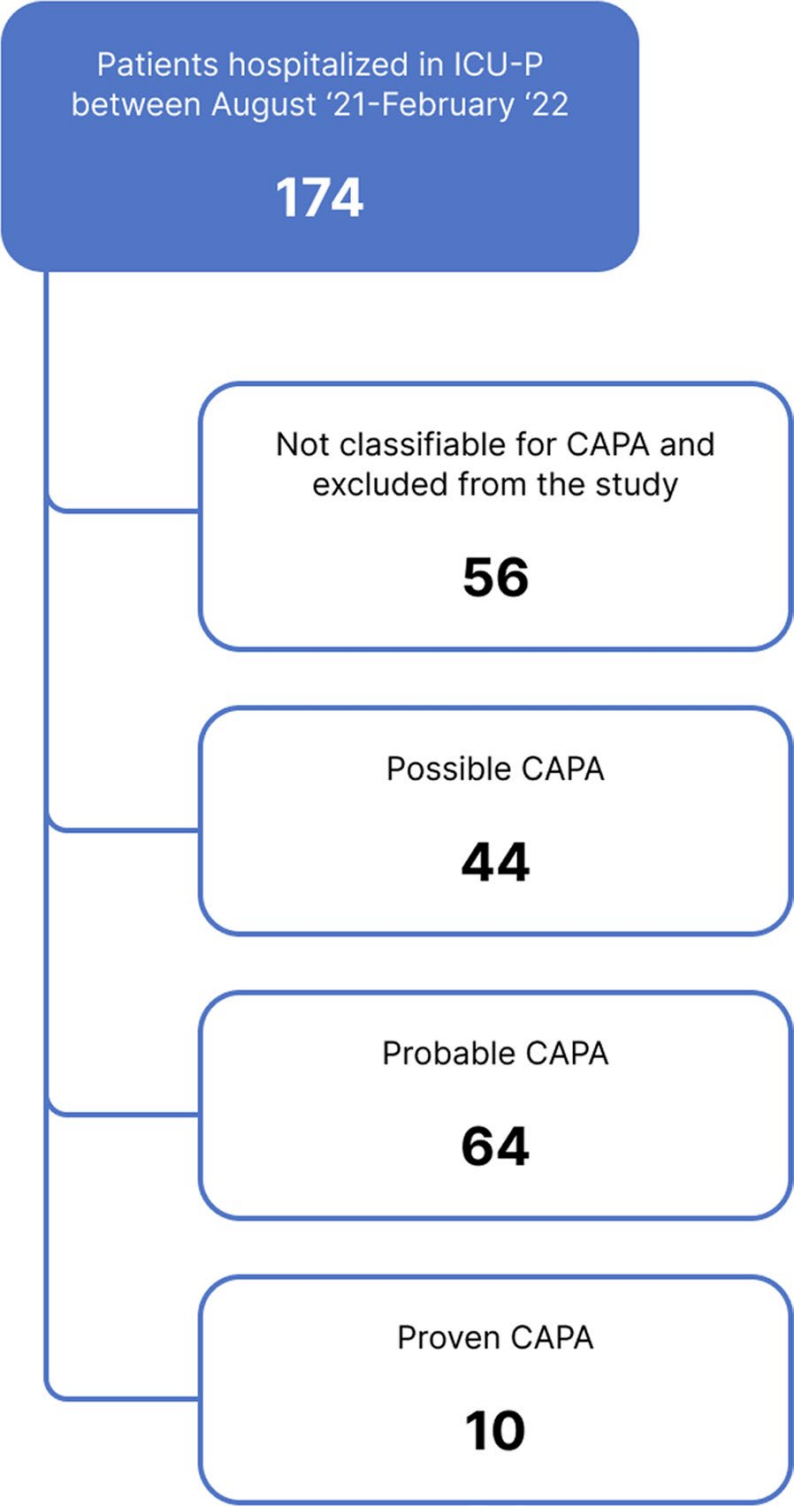
Patients’ flowchart. *ICU-P* intensive care unit for the pandemic, *CAPA* COVID-19-associated pulmonary aspergillosis

This study included patients with a diagnosis of COVID-19 cases confirmed by polymerase chain reaction (PCR) and were followed up in ICU-P between August 2021 and February 2022 with acute respiratory failure (ARF). The diagnosis of CAPA was based on the European Confederation of Medical Mycology (ECMM) and the International Society for Human and Animal Mycology 2020 (ECMM/ ISHAM) guidelines [5]. The criteria are shown in Table 1. Patients not considered for CAPA were excluded from the study. The remaining cases were categorized according to the relevant guideline (Fig. 1 Patients' flowchart).

**Table 1:** Diagnostic criteria for COVID-19-associated pulmonary aspergillosis (CAPA) according to ECMM/ ISHAM [5]

	Host factors	Clinical factors	Mycological evidence
Tracheobronchitis or other pulmonary forms (proven)	Patients with COVID-19 needing intensive care and a temporal relationship (entry criterion)		At least one of the following: Histopathological or direct microscopic detection of fungal hyphae, showing tissue damage; or aspergillus recovered by culture or microscopy or histology or PCR obtained by a sterile aspiration or biopsy
Tracheobronchitis (probable)	Patients with COVID-19 needing intensive care and a temporal relationship (entry criterion)	Tracheobronchitis, indicated by tracheobronchial ulceration, nodule, pseudomembrane, plaque, or eschar seen on bronchoscopic analysis	At least one of the following: Microscopic detection of fungal elements in bronchoalveolar lavage; positive bronchoalveolar lavage culture or PCR; serum galactomannan index > 0.5 or serum LFA index > 0.5; or bronchoalveolar lavage galactomannan index ≥ 1.0 or bronchoalveolar lavage LFA index ≥ 1.0
Other pulmonary forms (probable)	Patients with COVID-19 needing intensive care and a temporal relationship (entry criterion)	Pulmonary infiltrate, preferably documented by chest CT, or cavitating infiltrate (not attributed to another cause)	At least one of the following: Microscopic detection of fungal elements in bronchoalveolar lavage; positive bronchoalveolar lavage culture; serum galactomannan index > 0.5 or serum LFA index > 0.5; bronchoalveolar lavage galactomannan index ≥ 1.0 or bronchoalveolar lavage LFA index ≥ 1.0; two or more positive aspergillus PCR tests in plasma, serum, or whole blood; a single positive aspergillus PCR in bronchoalveolar lavage fluid
Other pulmonary forms (possible)	Patients with COVID-19 needing intensive care and a temporal relationship (entry criterion)	Pulmonary infiltrate, preferably documented by chest CT, or cavitating infiltrate (not attributed to another cause)	At least one of the following: Microscopic detection of fungal elements in non-bronchoscopic lavage; positive non-bronchoscopic lavage culture; single non-bronchoscopic lavage galactomannan index > 4.5; non-bronchoscopic lavage galactomannan index > 1.2 twice or more; or non-bronchoscopic lavage galactomannan index > 1.2 plus another non-bronchoscopic lavage mycology test positive

*CAPA* COVID-19-associated pulmonary aspergillosis, *COVID-19* Coronavirus-2019, *ECMM/ ISHAM* European Confederation of Medical Mycology (ECMM) and the International Society for Human and Animal Mycology 2020, *PCR* polymerase chain reaction, *CT* computed tomography, *LFA* lateral flow assay

**Fig. 2:**
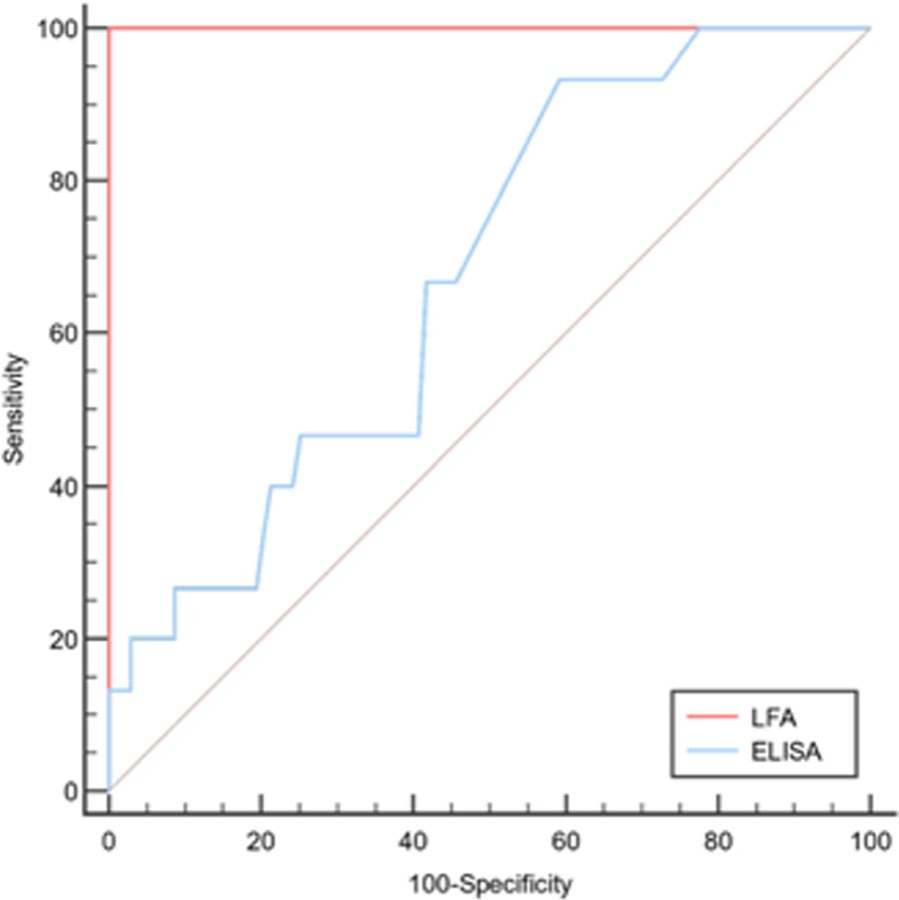
ROC curve for LFA and ELISA for the cut-off value of 0.5 ODI

**Fig. 3:**
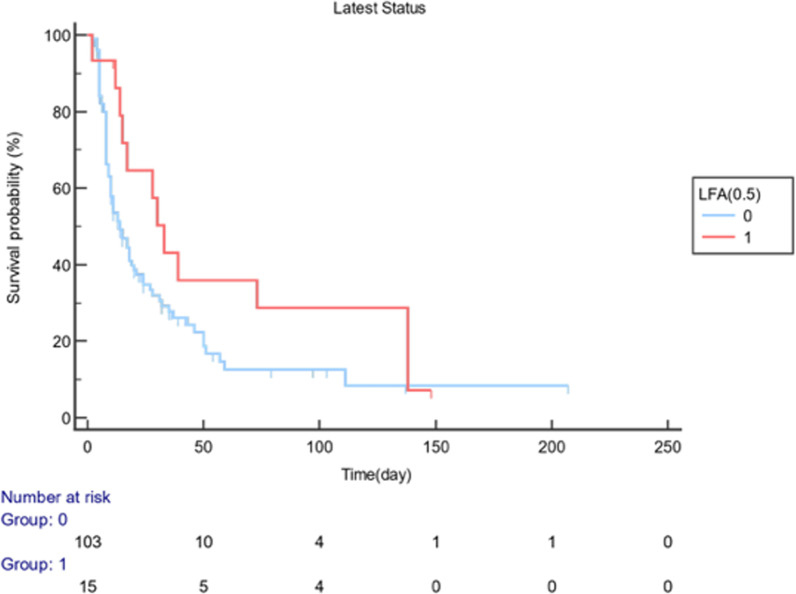
Probability of survival (%) vs. time: LFA for the cut-off value of 0.5 ODI

**Fig. 4:**
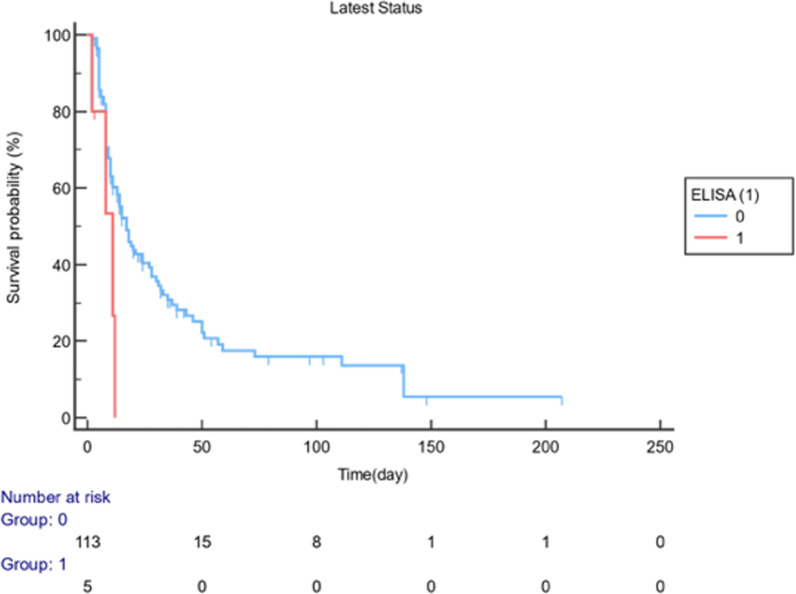
Probability of survival (%) vs. time: ELISA for the cut-off value of 1.0 ODI

In addition to demographic data such as age and gender, comorbidities, being diagnosed with an active solid or hematological malignancy, immunosuppressive drug usage, and survival of the patients included in the study were recorded. Galactomannan levels were determined using LFA and ELISA in serum samples taken simultaneously from the patients.

Serum samples were taken from all patients at the time of admission to the ICU-P and the results were obtained and recorded after studying the samples with LFA and ELISA on the same day. Sterile respiratory tract samples taken at the time of hospitalization or during follow-up in ICU-P were subjected to direct microscopic examination, culture on Sabouraud medium and cytopathological examination.

### LFA analysis

For the LFA (sōna *Aspergillus* galactomannan LFA, IMMY, Norman, Oklahoma, United States of America), 300 µL of serum was pretreated by addition of 100 µL of EDTA-containing buffer, heating at 120 °C for 6–8 min and centrifugation. Eighty microliters of the resulting supernatant were transferred to a separate test tube to which 40 μL of running buffer was added. A test strip was then inserted into this tube. Results were recorded after 30 min. All tests were performed by the same researcher (IS) and read using a digital reader (Cube reader, Chembio Diagnostics GmbH) provided by the manufacturer.

### Statistical analysis

In the analysis of the data, the mean and standard deviation, median, minimum and maximum values of the features, frequency, and percentage values were used to identify categorical variables. Parametric tests were used without the normality test due to the compatibility of the Central Limit Theorem [21]. A Chi-square test statistic was used to evaluate the relationship between two independent variables. In evaluating the diagnostic performance of the LFA and ELISA method, sensitivity, specificity, positive predictive value (PPV), negative predictive value (NPV), positive likelihood ratio, negative likelihood ratio, accuracy, and diagnostic odds ratio statistics were used. Differences based on LFA (cut-off: 0.5) and ELISA (cut-off: 1.0) groups were determined by the Log-Rank test, and the Hazard ratio (HR) was given with a 95% confidence interval. The statistical significance level of the data was taken as p < 0.05. New York software and MedCalc statistical package program were used for the data evaluation.

Ethical committee approval was received (Istanbul Training and Research Hospital, approval date, and number: 28/01/2022-28).

## Results

Out of the 174 patients followed in the ICU-P, 56 did not meet any criteria for CAPA and were excluded from the analysis. The rate of patients diagnosed with proven CAPA was 5.7% (10 patients). The most common comorbidity encountered was hypertension, with 32.2%. A total of 88 patients died in the ICU-P follow-up (74.6%) (Table 2).

**Table 2:** Descriptive statistics, demographic characteristics, clinical and biochemistry results of the patients

	x̄ ± SD	Median (Min–Max)
Features		
Age	67 ± 16.9	70.5 (18–92)

	n	%
*Gender*		
Female	50	42.4
Male	68	57.6
*HT*		
(−)	80	67.8
( +)	38	32.2
*DM*		
(−)	96	81.4
( +)	22	18.6
*CAD*		
(−)	105	89
( +)	13	11
*COPD*		
(−)	111	94.1
( +)	7	5.9
*Active malignancy*		
(−)	100	84.7
( +)	18	15.3
*Hematological malignancy*		
(−)	105	89
( +)	13	11
*Immunosuppressive usage*		
(−)	102	86.4
( +)	16	13.6
*Proven CAPA*		
(−)	108	91.5
( +)	10	8.5
*LFA (cut-off: 0.5)*		
(−)	103	87.3
( +)	5	12.7
*LFA (cut-off: 1.0)*		
(–)	116	98.3
( +)	2	1.7
*ELISA (cut-off: 0.5)*		
(−)	107	90.7
( +)	11	9.3
*ELISA (cut-off: 1.0)*		
(−)	113	95.8
( +)	5	4.2
*Last status*		
Alive	30	25.4
Exitus	88	74.6

	x̄ ± SD	Median (Min–Max)
Follow-up (days)	26.82 ± 35.8	13 (2–207)

*SD* standard deviation, *HT* hypertension, *DM* diabetes mellitus, *CAD* coronary artery disease, *COPD* chronic obstructive pulmonary disease, *CAPA* COVID-19-associated pulmonary aspergillosis, *LFA* lateral flow assay, *ELISA* enzyme-linked immunoassay

A statistically significant result was obtained with LFA for the cut-off value of 0.5 ODI in the diagnosis of CAPA (p < 0.001). The same significant statistical relationship was found for the cut-off value of 1.0 ODI for the ELISA (p < 0.01) (Table 3).

**Table 3:** Diagnostic performance of LFA and ELISA for proven CAPA: cut-off values of 0.5 and 1.0 ODI

Proven CAPA	(−) (n = 108)	( +) (n = 10)	p-value
	n (%)	n (%)	
*LFA (cut-off: 0.5)*			
(−)	101 (93.5)	2 (20)	** < 0.001**
(+)	7 (6.5)	8 (80)	
*LFA (cut-off: 1.0)*			
(−)	106 (98.1)	10 (100)	0.66
(+)	2 (1.9)	–	
*ELISA (cut-off: 0.5)*			
(−)	99 (91.7)	8 (80)	0.22
(+)	9 (8.3)	2 (20)	
*ELISA (cut-off: 1.0)*			
(−)	105 (97.2)	8 (80)	**0.01**
(+)	3 (2.8)	2 (20)	

Chi-square test (significance p < 0.05)

*LFA* lateral flow assay, *ELISA* enzyme-linked immunoassay, *ODI* optical density index, *CAPA* COVID-19-associated pulmonary aspergillosis

Table 4 shows the diagnostic test performance analysis for the cut-off value of 0.5 ODI for the LFA test in the diagnosis of CAPA. The sensitivity was 80% (95% CI: 0.55–1.05, p < 0.05) and the specificity was 94% (95% CI: 0.89–0.98, p < 0.05). Figure 2 shows the comparative ROC analysis of LFA and ELISA for the cut-off value of 0.5 ODI.

**Table 4:** Diagnostic test performance statistics of LFA for the cut-off value of 0.5 and diagnostic test performance statistics of ELISA for the cut-off value of 1.0 ODI

		95% Confidence interval lower	95% Confidence interval upper	p-value
*LFA (cut-off value: 0.5)*				
Sensitivity	0.8	0.55	1.05	** < 0.05**
Specificity	0.94	0.89	0.98	** < 0.05**
PPV	0.53	0.28	0.79	> 0.05
NPV	0.98	0.95	1.007	** < 0.05**
PLR	12.34	5.65	26.94	** < 0.05**
NLR	0.21	0.06	0.74	** < 0.05**
Accuracy	0.92	0.88	0.97	** < 0.05**
Odds ratio	57.71	10.25	325.07	** < 0.05**
*ELISA (cut-off value:1.0)*				
Sensitivity	0.2	− 0.05	0.45	> 0.05
Specificity	0.97	0.94	0.99	** < 0.05**
PPV	0.4	− 0.3	0.83	> 0.05
NPV	0.93	0.88	0.98	** < 0.05**
PLR	7.2	1.36	38.16	** < 0.05**
NLR	0.83	0.61	1.12	** < 0.01**
Accuracy	0.91	0.85	0.96	** < 0.05**
Odds ratio	8.75	1.27	60.18	** < 0.05**

*LFA* lateral flow assay, *ODI* optical density index, *PPV* positive predictive value, *NPV* negative predictive value, *PLR* positive likelihood ratio, *NLR* negative likelihood ratio

Table 4 shows also the diagnostic test performance analysis for the cut-off value of 1.0 ODI for the ELISA in the diagnosis of CAPA. The sensitivity was 20% (95% CI: 0.05–0.45, p > 0.05) and the specificity was 97% (95% CI: 0.94–0.99, p < 0.05).

The total number of patients who died in the follow-up interval of the 0-207^th^ day was 88 (74.58%), and the number of non-ex was 30 (25.42%). The results of the log-rank test for comparing the two survival curves are shown in Table 5. Life curves were found to differ substantially, and an LFA result of 0.5 ODI significantly affected survival (p < 0.05). The risk of death was 1.66 (HR: 1.66, 95% CI: 1.02–2.86, p < 0.05) times higher in patients with an LFA result of ≥ 0.5 ODI than those with < 0.5 (p < 0.05). Moreover, the risk of death was 18.01 (HR: 18.01, 95% CI: 1.39–46.24, p < 0.05) times higher in those with an ELISA result of ≥ 1.0 ODI than those with < 1.0 (p < 0.05) (Table 5).

**Table 5:** Comparison of survival curves (Log-Rank test) and hazard ratios with 95% confidence interval

	Exitus N (%)	Non-exitus N (%)	Median Survival (95% CI)	Hazard Ratio (95% CI)	Log-Rank p-value
*LFA (cut-off: 0.5)*					
( +) (n:15)	13(86.67)	2(13.3)	14(10–19)	1.66(1.02–2.86)	**0.05**
(−) (n:103)	75(72.82)	28(27.18)	33(14–138)		
Overall	88(74.58)	30(25.42)	17(11–24)		
*ELISA (cut-off: 1.0)*					
( +) (n:5)	4(80)	1(20)	11(2–12)	8.01(1.39–46.24)	**0.02**
(−) (n:113)	84(74.34)	29(25.66)	17(13–24)		
Overall	88(74.58)	30(25.42)	17(11–24)		

*CI* confidence interval, *LFA* lateral flow assay, *ELISA* enzyme-linked immunoassay

In Fig. 3, it was observed that the study was terminated on day 207 and the probability of survival (%) decreased. “Number at risk” indicates the number of patients at risk at the end of each period. While 15 patients with LFA results of ≥ 0.5 ODI were at risk at the beginning of the study (Day 0), the number of patients at risk was 5 at the end of the 50th day. In Fig. 4, the same analysis was performed for the ELISA cut-off value of ≥ 1.0 ODI. At the beginning of the study (Day 0), 5 patients were at risk, while at the end of the 50th day, the number of patients at risk was 0.

## Discussion

This study reveals new findings in many aspects. While there were significant statistical results with LFA for the cut-off value of 0.5 ODI in the diagnosis of CAPA, the same significant statistical relationship was found for the cut-off value of 1.0 ODI for the ELISA. For the cut-off value of 0.5 ODI, sensitivity was 80%, specificity 94%, PPV 53% and NPV 98% for LFA. In addition, the risk of death was 1.66 times higher in patients with an LFA result of ≥ 0.5 ODI than those with < 0.5. LFA could play an important role in diagnosing aspergillosis secondary to viral infections due to its high sensitivity–specificity and ease of administration.

A review from 2022 revealed that the incidence of CAPA in patients with ICU follow-up ranges from 0 to 34.4% [22]. In another study, the incidence of CAPA was 10–15% in patients with ICU follow-up, and mortality was reported to be between 43 and 52% [7]. In another review, 1421 patients were evaluated, and CAPA mortality was found to be 48.4% [23]. In our study, the rate of patients diagnosed with proven CAPA was 5.7%, while mortality was 74.6% despite all effective treatment options. It should be noted that our center serves a large number of immigrant patients and therefore the number of patients who are not under follow-up and treatment in terms of their comorbidities caused the mortality rate to be quite high compared to other studies in literature.

In a recent study from 2022 [24], LFA was evaluated in COVID-19 patients with ARF; while the sensitivity of LFA was 20% for the cut-off value of 0.5 ODI, the specificity was 93%. The sensitivity for the cut-off value of 1.0 ODI was 9%, while the specificity was 99%. In this study, the diagnostic performance was examined with the combination of respiratory samples. In the analysis performed by combining tracheal aspirate, non-directed bronchial lavage, and bronchoalveolar lavage samples, the sensitivity was 83%, and the specificity was 44% for the cut-off value of 0.5 ODI. For the cut-off value of 1.0 ODI, the sensitivity was 81%, and the specificity was 67%. In another study [25], galactomannan antigen was studied by LFA and ELISA in both serum and bronchoalveolar lavage samples of COVID-19 patients followed up in the ICU.The sensitivity of LFA in serum samples for 0.5 ODI turned out to be 56.3% while the specificity was 94.2%. In our study, the sensitivity was 80%, and the specificity was 94% for the cut-off value of 0.5 ODI for LFA. The same statistical relationship could not be found for the cut-off value of 1.0 ODI for the LFA contrary to ELISA. Therefore, it is suggested that LFA can be used with very high sensitivity and specificity for 0.5 ODI in suspicion of CAPA. LFA seems to be more successful compered to ELISA in terms of low galactomannan-antigenemia.

Serum LFA efficacy has been studied in hematological malignancies where invasive fungal infections are common, and its efficacy has also been demonstrated. In the study of Hoenigl et al. [26], sensitivity for the cut-off value of 0.5 ODI was 78.6%, while specificity was 80.5%. In the study conducted in our clinic [20], the sensitivity was 90.9%, and the specificity was 90.8% for the same cut-off value. Although studies on the effectiveness of serum LFA in hematological malignancies seem to be more compatible with each other, CAPA has emerged as a newer field in evaluating the effectiveness of the LFA test. In our study, LFA yields successful results with a sensitivity of 80% and a specificity of 94% for 0.5 ODI for the diagnosis of CAPA. It is a non-invasive, rapid and effective diagnostic method for 0.5 ODI and can play an important role in the early diagnosis and treatment of ICU-patients for whom invasive diagnostic methods may not seem to be favorable options.

Besides diagnosing CAPA with high sensitivity and specificity, our study revealed important data regarding mortality. The risk of death was 1.66 times higher in patients with an LFA result of ≥ 0.5 ODI than those with < 0.5. In the multivariate analysis performed in the MYCOVID study from 2022, which included patients also with ICU follow-up, it was revealed that the diagnosis of probable or possible CAPA increased the mortality risk 1.45 times (HR: 1.45, 95% CI: 1.03–2.03, p = 0.033) [27]. Within current practices, where insufficient awareness is an important factor in the diagnosis and early treatment of CAPA, it could be thought that LFA will play an important role in high diagnostic performance and efficacy in mortality prediction.

There were also some limitations of this study. The fact that interventional methods are less accessible due to pandemic conditions may have limited the “proven” patient group. The fact that PCR is not used to diagnose *aspergillus* in our clinic might also have limited the proven CAPA group.

## Conclusions

In conclusion, for LFA, the sensitivity was 80%, the specificity 94%, the PPV 53%, and NPV was 98% for the cut-off value of 0.5 ODI and was found to be superior to ELISA. In addition, the risk of death was 1.66 times higher in patients with an LFA result of ≥ 0.5 ODI than those with < 0.5. Concerning its efficiency in patients with hematological malignancies and accuracy diagnosing CAPA, LFA expected to be a useful and effective part of future clinical practices.

## Data Availability

Datasets analyzed during the current study are available in [Mendeley Data] [https://doi.org/10.17632/vc8rxy598m.1].

## Abbreviations

COVID-19: Coronavirus-2019
IPA: Invasive pulmonary aspergillosis
CAPA: COVID-19-associated pulmonary aspergillosis
ICU: Intensive care units
ELISA: Enzyme-linked immunoassay
LFA: Lateral flow assay
ODI: Optical density index
ICU-P: Intensive care unit for pandemic
PCR: Polymerase chain reaction
ARF: Acute respiratory failure
ECMM/ISHAM: European Confederation of Medical Mycology (ECMM) and the International Society for Human and Animal Mycology 2020
PPV: Positive predictive value
NPV: Negative predictive value
HR: Hazard ratio
